# The world according to zebrafish: how neural circuits generate behavior

**DOI:** 10.3389/fncir.2014.00091

**Published:** 2014-07-30

**Authors:** Germán Sumbre, Gonzalo G. de Polavieja

**Affiliations:** ^1^Ecole Normale Supérieure, Institut de Biologie de l'ENSParis, France; ^2^Inserm, U1024Paris, France; ^3^CNRS, UMR 8197Paris, France; ^4^Instituto Cajal, Consejo Superior de Investigaciones CientíficasMadrid, Spain; ^5^Champalimaud Neuroscience Programme, Champalimaud Center for the UnknownLisbon, Portugal

**Keywords:** zebrafish, behavior, neuronal circuit dynamics, neuroanatomy, Models of human brain disorders

Understanding how the brain controls motor behavior and generates cognitive functions still remains one of the most challenging goals in science and neuroscience in particular.

Toward this goal it is important to use a multidisciplinary approach involving genetics, molecular biology, optics, ethology, neurobiology, and mathematical modeling. This strategy is most efficient when using animal models with relatively simple nervous systems still capable of producing complex motor behaviors. Genetically tractable models enable labeling specific neurons and monitoring and manipulating neuronal activity of single cells or entire circuits via optogenetics (Fenno et al., 2011; Akerboom et al., 2013; Aston-Jones and Deisseroth, 2013; Chen et al., 2013; Marvin et al., 2013).

The zebrafish *Danio rerio* is a small shoaling tropical fresh-water fish native to rivers of south Asia. It is a member of the teleostei infraclass, a monophyletic group that emerged ~340 million years ago (Amores et al., 2011). Compared to other vertebrate species, teleost fish underwent an additional round of whole-genome duplication (Meyer and Schartl, 1999).

Zebrafish has been used for developmental and genetic studies since the late 1950s. By the 1980s, zebrafish was already used as a genetically tractable organism. In 2001 the zebrafish genome-sequencing project was launched and recently its protein-coding genes were compared to those of humans (Friedrich et al., 2010; Howe et al., 2013). This large-scale project showed that zebrafish have 26,206 protein-coding genes (Collins et al., 2012), with ~70% of human genes having at least one obvious zebrafish ortholog (Howe et al., 2013).

The combination of high-throughput mutagenesis and TILLING (Wienholds et al., 2003) or specifically targeted DNA sequence mutations [Zinc-finger nucleases (Doyon et al., 2008), TALENS (Sander et al., 2011) and CRISPR (Hwang et al., 2013)], enable DNA precise editing and thus the generation of transgenic and/or specific mutant zebrafish lines. Among this increasing collection of available mutants, several were identified as vertebrate models of certain human neurodevelopmental, neurological, and neurodegenerative syndromes and diseases [e.g., Parkinson's (Lam et al., 2005; Flinn et al., 2008), Alzheimer's (Newman et al., 2007, 2011), Rett's syndrome (Pietri et al., 2013), ALS (Gibbs et al., 1976; Burrill and Easter, 1994; Da Costa et al., 2014), tinnitus (Wu et al., 2014), psychiatric disorders (Norton, 2013), Huntington's disease (Schiffer et al., 2007), Lowe's syndrome (Ramirez et al., 2012), and more (Sager et al., 2010)].

Furthermore, large-scale enhancer-trap screens in combination with DNA insertion methods (e.g., Tol2, Kawakami and Shima, 1999), bioinformatics and the Gal4/UAS system generated a vast collection of transgenic fish and a large database of tissue/cell-type specific promoters (Scott et al., 2007; Asakawa et al., 2008).

An additional advantage of the zebrafish larva model is its transparent skin, small size and the fact that it mainly uses cutaneous breathing (up to ~14 days post-fertilization, dpf). These three characteristics make possible to restrain larvae in a drop of low-melting agarose without the use of any paralyzers or anesthetics, in intact conditions, without the use of surgical procedures to expose and image the brain.

With the development of recent state-of-the-art optical techniques including two-photon scanning microscopy (Ahrens et al., 2012; Portugues et al., 2014), Single plain illumination microscopy (Ahrens et al., 2013b; Panier et al., 2013), lightfield microscopy (Broxton et al., 2013), and Spatial light modulator microscopy (Quirin et al., 2013), the entire brain can be now simultaneously imaged and its activity monitored with single or near single-cell resolution. On the other hand, fiber optics (Miri et al., 2011; Kubo et al., 2014), Digital micromirror devices (Wyart et al., 2009), and holographic pattern illumination (Vaziri and Emiliani, 2012) can be used to stimulate optogenetic tools in single cells or large neuronal circuits.

The combination of all these techniques together with the larva's small size and skin transparency enable monitoring in toto brain dynamics and manipulate its activity in an intact, non-anesthetized, non-paralyzed vertebrate (Ahrens et al., 2013b; Panier et al., 2013; Portugues et al., 2014).

From a behavioral point of view, upon hatching the larva needs to immediately catch prey and avoid predators in order to survive. This strong evolutionary pressure leads to a rapid development of functional sensory systems in general, and vision in particular, creating a reach repertoire of visuo-motor behaviors (Fleisch and Neuhauss, 2006; Portugues and Engert, 2009). For example, the startle escape response [a flash of light induces a directional swimming behavior (Burgess and Granato, 2007)], optokinetic response [OKR, compensatory eye saccades evoked by coherent field motion (Rinner et al., 2005; Mueller and Neuhauss, 2010)], optomotor response [OMR, compensatory tail movements evoked by coherent field motion (Orger et al., 2000)], dorsal-light response (DLR, Neuhauss, 2003), feeding behavior (Budick and O'Malley, 2000), and eye lateralization [preferential use of one eye over the other depending on the type of visual stimulus (Miklósi and Andrew, 2006)]. Furthermore, they also show rheotaxis (Olszewski et al., 2012), odor and gustatory-induced behaviors (Mathuru et al., 2012; Boyer et al., 2013), learning and memory (Aizenberg and Schuman, 2011; Valente et al., 2012; Roberts et al., 2013), and circadian rhythms and sleep (Naumann et al., 2010; Chiu and Prober, 2013; Elbaz et al., 2013), among others.

At the juvenile and adult stages, zebrafish develop more complex behaviors such as social learning, shoaling, group decision making and learning, courtship, territoriality, and hierarchy (Arganda et al., 2012; Oliveira, 2013).

With all these multidisciplinary advantages in hand and a relative simple nervous system [~100,000 neurons at 7 dpf (Hill et al., 2003; Naumann et al., 2010)], still with a well-conserved vertebrate structure, the zebrafish is becoming an emerging experimental model in neurosciences and neuroethology (Friedrich et al., 2010).

Recent studies have shown that it is also possible to monitor and/or manipulate neuronal dynamics in partially agarose-restrained behaving intact larvae and therefore correlate neuronal activity and motor behavior. Moreover, close-loop visual virtual reality can be used so larvae can get visual feedback of their own acts despite being immobilized (Ahrens et al., 2012, 2013a).

Alternatively, although lacking cellular resolution, using transgenic larvae expressing a bioluminescence protein such as GFP-Aequorin expressed in specific cell populations, it is possible to monitor brain activities in unrestrained freely behaving animals (Naumann et al., 2010).

Furthermore, due to the zebrafish ex-uterus development all embryonic and larval developmental stages following fecundation are accessible for imaging.

The combination of the genetic and optical state-of-the-art techniques with the zebrafish experimental model is yielding high-dimensional large data sets pushing the limits of current data analysis standards, forcing for the development of new methodologies and novel theoretical models.

A future challenge in the field will be monitoring whole-brain activity with near single-neuron resolution from multiple freely behaving and socially interacting individuals.

In this topic we have gathered a collection of original articles, reviews, and opinions covering a wide-spectrum of topics from behavior up to whole-brain activity recordings, both in wild type and in neurological human-syndrome models, providing an overview of current state and future directions of zebrafish circuits neuroscience and behavior research field.

We have organized this eBook in 4 different chapters:

1. Neuroanatomy
2. Neuronal circuit dynamics
3. Behavior
4. Models of brain disorders and addiction

The zebrafish model in combination with recently developed imaging techniques, optogenetics, and sophisticated mathematical methods for analysis of the acquired large data sets is bringing us closer than ever before to the understanding of how brain dynamics relates to behavior.

## Conflict of interest statement

The authors declare that the research was conducted in the absence of any commercial or financial relationships that could be construed as a potential conflict of interest.
